# Supplementary material for: IoT-CCAC: a blockchain-based consortium capability access control approach for IoT
Source: PeerJ Comput Sci. 2021 Apr 8;7:e455. doi: 10.7717/peerj-cs.455 (PMC8049119; doi:10.7717/peerj-cs.455)
Supplement: Supplemental Information 2 [file peerj-cs-07-455-s002.zip › CCapAC-master/CCapAC/admin/templates/admin.html]

{% extends 'base.html' %}
{% block content %}

## Assets Table

The table presents all the resources in the system

| Asset ID | Issuer | Date issue | Owner | Resource ID | Type | Resource URI | Resource Func |
| --- | --- | --- | --- | --- | --- | --- | --- |
{% for asset in data.assets %}| {{asset.context.uid}} | \*\*\*\* | {{asset.context.date}} | {{asset.entityCredential.owner}} | {{asset.entityCredential.id}} | {{asset.entityCredential.type}} | {{asset.entityCredential.uri}} | {{asset.entityMetadata.func}} |
{% endfor %}

---

## Services Table

The table presents all the services in the system

| Service ID | Service name | Issuer | Date issue | Service init | SM number | Request quota |
| --- | --- | --- | --- | --- | --- | --- |
{% for service in data.services %}| {{service.context.id}} | {{service.serviceCredential.name}} | \*\*\*\* | {{service.context.date}} | {{service.serviceCredential.service\_init}} | {{service.metadata.sm\_number}} | {{service.metadata.req\_quota}} |
{% endfor %}

---

## Profiles Table

The table presents all the profiles in the system

| Profile ID | Asset ID | Service ID | Date issue | Issuer |
| --- | --- | --- | --- | --- |
{% for profile in data.profiles %}| {{profile.context.id}} | {{profile.profileCredential.asset\_id}} | {{profile.profileCredential.service\_id}} | {{profile.context.date}} | \*\*\*\* |
{% endfor %}

{% endblock %}
