# Supplementary material for: IoT-CCAC: a blockchain-based consortium capability access control approach for IoT
Source: PeerJ Comput Sci. 2021 Apr 8;7:e455. doi: 10.7717/peerj-cs.455 (PMC8049119; doi:10.7717/peerj-cs.455)
Supplement: Supplemental Information 2 [file peerj-cs-07-455-s002.zip › CCapAC-master/CCapAC/admin/templates/assetform.html]

{% extends 'base.html' %}
{% block content %}

Domains

www.example.com/v1/org1
www.example.com/v1/org2
www.example.com/v1/org3

GenKey

Types

Sensor
Actuator
Device

Entity URI

Functions

Temperature
Proximity
Light
Pressure
Smoke

Add asset

{% endblock %}
