# Supplementary material for: IoT-CCAC: a blockchain-based consortium capability access control approach for IoT
Source: PeerJ Comput Sci. 2021 Apr 8;7:e455. doi: 10.7717/peerj-cs.455 (PMC8049119; doi:10.7717/peerj-cs.455)
Supplement: Supplemental Information 2 [file peerj-cs-07-455-s002.zip › CCapAC-master/CCapAC/admin/templates/bigchaindb.html]

{% extends 'base.html' %}
{% block content %}

## Assets BigDB

The table presents all the resources in the BigchainDB

| ID | Prepare Start | Prepare End | Fulfill Start | Fulfill End | Commit Start | Commit End |
| --- | --- | --- | --- | --- | --- | --- |
{% for asset in data.assets %}| {{asset.BigDB\_id}} | {{asset.prepar\_time.start}} | {{asset.prepar\_time.finish}} | {{asset.fulfill\_time.start}} | {{asset.fulfill\_time.finish}} | {{asset.commit\_time.start}} | {{asset.commit\_time.finish}} |
{% endfor %}

---

## Services BigDB

The table presents all the services in the BigchainDB

| ID | Prepare Start | Prepare End | Fulfill Start | Fulfill End | Commit Start | Commit End |
| --- | --- | --- | --- | --- | --- | --- |
{% for service in data.services %}| {{service.BigDB\_id}} | {{service.prepar\_time.start}} | {{service.prepar\_time.finish}} | {{service.fulfill\_time.start}} | {{service.fulfill\_time.finish}} | {{service.commit\_time.start}} | {{service.commit\_time.finish}} |
{% endfor %}

---

## Profiles BigDB

The table presents all the profiles in the BigchainDB

| ID | Prepare Start | Prepare End | Fulfill Start | Fulfill End | Commit Start | Commit End |
| --- | --- | --- | --- | --- | --- | --- |
{% for profile in data.profiles %}| {{profile.BigDB\_id}} | {{profile.prepar\_time.start}} | {{profile.prepar\_time.finish}} | {{profile.fulfill\_time.start}} | {{profile.fulfill\_time.finish}} | {{profile.commit\_time.start}} | {{profile.commit\_time.finish}} |
{% endfor %}

---

## Statements BigDB

The table presents all the statements in the BigchainDB

| ID | Prepare Start | Prepare End | Fulfill Start | Fulfill End | Commit Start | Commit End |
| --- | --- | --- | --- | --- | --- | --- |
{% for statement in data.statements %}| {{statement.BigDB\_id}} | {{statement.prepar\_time.start}} | {{statement.prepar\_time.finish}} | {{statement.fulfill\_time.start}} | {{statement.fulfill\_time.finish}} | {{statement.commit\_time.start}} | {{statement.commit\_time.finish}} |
{% endfor %}

{% endblock %}
