# Supplementary material for: IoT-CCAC: a blockchain-based consortium capability access control approach for IoT
Source: PeerJ Comput Sci. 2021 Apr 8;7:e455. doi: 10.7717/peerj-cs.455 (PMC8049119; doi:10.7717/peerj-cs.455)
Supplement: Supplemental Information 2 [file peerj-cs-07-455-s002.zip › CCapAC-master/CCapAC/admin/templates/profileform.html]

{% extends 'base.html' %}
{% block content %}

Assets

{% for asset in data.assets %}
{{ asset.context.uid }}- {{ asset.entityCredential.type }} - {{ asset.entityMetadata.func }} - {{ asset.entityCredential.owner }}
{% endfor %}

Services

{% for service in data.services %}
{{service.serviceCredential.name}} - {{service.serviceCredential.service\_init}}
{% endfor %}

Create profile

{% endblock %}
