# Supplementary material for: IoT-CCAC: a blockchain-based consortium capability access control approach for IoT
Source: PeerJ Comput Sci. 2021 Apr 8;7:e455. doi: 10.7717/peerj-cs.455 (PMC8049119; doi:10.7717/peerj-cs.455)
Supplement: Supplemental Information 2 [file peerj-cs-07-455-s002.zip › CCapAC-master/CCapAC/admin/templates/statements/base.html]

{% if title %}
{{ title }}
{% else %}
Barber App
{% endif %}


{% if 'username' not in session %}- Login
{% else %}- Admin Home
- Manage Statements
- Tokens
- Create Statement
- Logout
{% endif %}


{% with messages = get\_flashed\_messages(with\_categories=true) %}
{% if messages %}
{% for category, message in messages %}

{{ message }}

{% endfor %}
{% endif %}
{% endwith %}
{% block content %}{% endblock %}
