# Supplementary material for: IoT-CCAC: a blockchain-based consortium capability access control approach for IoT
Source: PeerJ Comput Sci. 2021 Apr 8;7:e455. doi: 10.7717/peerj-cs.455 (PMC8049119; doi:10.7717/peerj-cs.455)
Supplement: Supplemental Information 2 [file peerj-cs-07-455-s002.zip › CCapAC-master/CCapAC/admin/templates/statements/statement.html]

{% extends 'statements/base.html' %}
{% block content %}

## Statements Table

The table presents all the statements in the system

| SID | Issuer | Date issue | Principle | Profile ID | Action | Resource URI |
| --- | --- | --- | --- | --- | --- | --- |
{% for statement in data.statements %}| {{statement.context.sid}} | \*\*\*\* | {{statement.context.date}} | {{statement.context.principal}} | {{statement.statementCredential.profile\_id}} | {{statement.statementCredential.action}} | {{statement.statementCredential.uri}} |
{% endfor %}

{% endblock %}
