# Supplementary material for: IoT-CCAC: a blockchain-based consortium capability access control approach for IoT
Source: PeerJ Comput Sci. 2021 Apr 8;7:e455. doi: 10.7717/peerj-cs.455 (PMC8049119; doi:10.7717/peerj-cs.455)
Supplement: Supplemental Information 2 [file peerj-cs-07-455-s002.zip › CCapAC-master/CCapAC/admin/templates/statements/statementform.html]

{% extends 'statements/base.html' %}
{% block content %}

Services

Choose a service
{% for service in data.services %}
{{service.serviceCredential.name}}
{% endfor %}

Profiles

Actions

Read
Write
Read & Write

Create statement

{% endblock %}
