# Supplementary material for: IoT-CCAC: a blockchain-based consortium capability access control approach for IoT
Source: PeerJ Comput Sci. 2021 Apr 8;7:e455. doi: 10.7717/peerj-cs.455 (PMC8049119; doi:10.7717/peerj-cs.455)
Supplement: Supplemental Information 2 [file peerj-cs-07-455-s002.zip › CCapAC-master/CCapAC/admin/templates/statements/tokens.html]

{% extends 'statements/base.html' %}
{% block content %}

## Token verification:

Send

{{ data.decode }}

---

## Statements Table

The table presents all the statements in the system

| statement ID | Token |
| --- | --- |
{% for token in data.tokens %}| {{token.id}} | {{token.token}} |
{% endfor %}

{% endblock %}
